# Workforce Perspectives of Sustaining the Utilisation of a Harm Reduction Instrument in a Mental Health Residential Setting

**DOI:** 10.3390/healthcare12020276

**Published:** 2024-01-22

**Authors:** Simon Kroes, Hannah McKim, Melissa Petrakis

**Affiliations:** 1 Mental Health Service, St Vincent’s Hospital Melbourne, Fitzroy 3065, Australia; simon.kroes@svha.org.au; 2 Social Work Department, School of Primary and Allied Health Care, Caulfield Campus, Monash University, Caulfield East 3145, Australia; hannah.mckim@monash.edu

**Keywords:** harm reduction, dual diagnosis, mental health, addiction, alcohol and other drugs, implementation

## Abstract

Purpose: This exploratory study investigated worker experiences of utilising the Before During After (BDA) harm reduction instrument to engage well with service users in a residential mental health service setting. Stakeholder interviews were conducted with a purposive sample of two senior nurses and one senior allied health staff at the study site to explore the impacts of BDA implementation on their work after 3 years of its use. A thematic analysis was conducted, including two-level coding. Five major themes were discussed. Of particular interest, and the focus of this paper, are the themes of effect on service users and effect on staff. The study found improved engagement between staff and service users, reduced stigma and more holistic care that was collaborative. In regard to staff, it was found that staff knowledge and confidence increased in addressing harm reduction issues with consumers and this was sustained over 3 years. Use of the BDA clinical instrument and package was reported to enhance worker engagement, knowledge and confidence in dual diagnosis work with service users.

## 1. Introduction

Internationally, alcohol and drug use cause considerable disease burden [1]. It has been suggested that psychoactive drug use accounts for over 400,000 deaths per annum [2], and others suggest the use of legal drugs far outweigh the impact of illegal ones on the global burden of disease [3]. According to the Australian Institute of Health and welfare in “2017–2018, nearly 4 in 5 Australians aged 18 and over drank alcohol in the past 12 months” and alcohol “was the only drug where approval of regular use by an adult was higher than disapproval” by more than double—“45% approved and 21% disapproved” [4]. More locally, the recent Royal Commission into Victoria’s Mental Health System stated “that mental and substance use ‘disorders’ were the second highest contributor to the overall age-standardised disease burden in Victoria in 2015. As the OECD has stressed, these costs should not be underestimated” [5].

Despite alcohol and drug use being classified as mental health issues under the major psychiatric classification systems of the DSM 5 [6] and ICD 10 [7], treatment of drug use, particularly alcohol, appears to be undertreated [8]. This could mean service users residing in mental health facilities may not be adequately treated for their alcohol and/or other drug use. Harm reduction could serve a useful purpose in such settings. Harm reduction includes policies, programs and practices with the primary aim to reduce adverse health, social and economic impacts or consequences associated with the use of both legal and illegal psychoactive drugs; while not necessarily reducing overall drug consumption [9]. Harm reduction has been established as robust and beneficial in mental health and alcohol and other drug use contexts [9,10].

The various tools available tend not to provide an integrated method of exploring dual diagnosis issues, despite a growing body of evidence suggesting integrated treatment is the best approach [10]. Or they are impractical to implement in terms of time taken to train staff or the time available to use them in busy clinical service settings. They are often aimed at the clinician as the person to gather evidence about the service user and then decide the best treatment. Yet, service user involvement in their own treatment is a crucial element of recovery and best practice [11].

A great tool is potentially only as good as its implementation, and this can be a difficult [12] and chaotic process [13]. Implementation is a fundamental issue. Some of this complexity can possibly be reduced by the use of user friendly, evidence-informed tools and associated implementation packages that have a quality improvement element. However, it seems few guidelines for clinical practice have been described regarding how harm reduction is actually practiced in dual diagnosis settings. This can limit the potential implementation of such approaches into day-to-day practice [14].

### 1.1. Study Aim

This study sought to discover the impact and sustainability of the use of a harm reduction instrument and associated implementation package on practice at a community care unit through semi-structured key informant interviews.

### 1.2. Research Question

What were the factors that facilitate or inhibit the uptake of an evidence-based treatment approach to dual diagnosis practice in routine care in a residential mental health treatment setting?

## 2. Materials and Methods

### 2.1. Research Design

To explore the impact of a harm reduction package on practice within a residential mental health service, a qualitative approach was chosen as most relevant for this study given its exploratory nature. In terms of a theoretical orientation, pragmatism was chosen to align with the nature of implementing change in a complex and dynamic service where learning by doing is essential [15]. Pragmatism is useful in undertaking practice-based research [16] because such research draws on pragmatic evidence-informed approaches, inclusive of the practice wisdom of practitioners [17]. Change, moving from practice as usual to one informed by harm reduction, is often dependent on the perspectives of those involved in the change, so it was fitting too for this study that the staff involved in this change process be interviewed and their perspectives described.

### 2.2. Study Setting

The study took place in Community Care Units (CCU) located in an inner-city area of Melbourne, Australia. There are 20 beds on site and medium- to long-term recovery and clinical services are available to eligible adult service users. Eligibility criteria include serious mental illness and related psycho social disabilities. Dual diagnosis, mental health issues and alcohol and/or other drug use, is a common issue self-reported by service users.

The capacity-building advisory service is auspiced by a public hospital in central Melbourne. One of four Metropolitan teams, the service works across 5 local government areas and with 2 large rural area partners. The service was established in 2001 as part of the Victorian Dual Diagnosis Initiative to enhance dual diagnosis capacity building in clinical mental health, mental health community support services (MHCSS) and alcohol and other drug (AOD) services. It is not a direct service provider; however, it supports a range of service provision organisations. Its role in this study was to assist the implementation of the BDA which included providing training and mentoring to staff as well as other implementation activities.

### 2.3. The BDA Clinical Instrument and Package

The BDA instrument was designed and developed by the first author in 1995 as a personal aid to treatment planning in relation to harm reduction. The idea was that by having a simple structure to use—a mnemonic—that interactions with service users about harm reduction could be conducted in a more holistic and consistent manner. It is now part of a workforce development package designed to assist staff to learn about harm reduction and provide them with an instrument and approach they can use with service users [18]. The BDA instrument, the central part of the package, provides a consistent structure for exploring harm reduction with service users. It prompts a service user-centred and recovery-focused collaborative process involving staff and service users, exploring harm reduction in relation to alcohol and/or other drug use and developing possible strategies and goals for reducing that harm and, perhaps more importantly, focusing on quality of life.

The BDA instrument aims to provide service users and staff with a user-friendly tool to guide collaborative treatment. There are other tools available to address alcohol and/or other drug issues. These include the Alcohol Use Disorders Identification Test [19], the Alcohol, Smoking and Substance Involvement Screening Test [20] and the Indigenous Risk Impact Screen [21]. However, where these differ from the BDA is that they are not as purposively collaborative nor do they have an integrated approach to alcohol and/or other drug use and mental health issues, dual diagnosis, together. It is believed that having an instrument for the purpose of exploring service user alcohol and/or other drug use and ways to reduce harm will increase staff knowledge and confidence in using harm reduction as part of their practice, in particular dual diagnosis practice. It may also have other benefits.

### 2.4. Ethical Considerations

Prior to recruitment, ethics approval was granted by the Research Ethics Committee (HREC-A) at the study hospital, and the University Human Research Ethics Committee (HREC). Participation was voluntary, with the right to withdraw from the study at any time. Participant confidentiality was ensured by de-identifying all transcripts and written feedback.

### 2.5. Participant Recruitment

A purposive sampling technique was used to select participants. There were three key staff at the service who were the only staff members involved at every stage of the project implementation, so they were approached. One of the staff members was a manager, one held the alcohol and other drugs portfolio for the service and was selected to champion the implementation, and one was a senior clinician. Participation in the current research study was voluntary. This was an unfunded study; no financial incentives were available to the participants. Key staff were invited via email to participate in the study, were provided with written consent forms and were informed that if they proceeded their participation was considered as consent. All three key informants agreed to participate.

### 2.6. Data Collection

Interview methods with semi-structured questions were selected for rich, layered discussion. Interview questions were informed by the literature review conducted by the first author. The interviews were conducted by the first author, a senior clinician in a practice change role (insider research), using a narrative approach so as to facilitate participants expressing views and opinions [22]. Initially, participants were to be interviewed in their workplaces but given the COVID-19 pandemic and ensuing restrictions in Victoria, Australia, interviews had to be conducted via an online video conferencing platform. One of the participants was home-schooling her children and was interrupted numerous times during the interview. Another had to do the interview from her car because there were issues with her internet connection in her usual workplace. The third was working from home at the time of the interview. Nevertheless, all participants completed a semi-structured interview of approximately one hour. Participants were asked about five areas. These were the effect on service users, effect on staff, effect on the service, evidence of sustained change and their personal contribution to the change process. These areas were developed through multiple conversations with staff involved in the implementation and co-authors. Areas of interest arose and formed the basis of the semi-structured interviews. Interviews were recorded and transcribed.

### 2.7. Data Analysis

Video recordings of the interviews were transcribed and analysed using an interpretive approach [15]. The steps in the approach were the following: The first-named author analysed transcribed raw data using first and second level coding [23,24], locating ‘the data into meaning units’ [24] into a table. Points of connection were then found within meaning units, such that they could be placed in categories until data became repetitive and saturation of categories was reached [14]. The themes that emerged were checked by the second-named author for content validity [24]. The language and expression used by participants have been included in the Results as direct quotes, included verbatim.

## 3. Results

The five themes discussed were the effect of using the BDA with service users, effect on staff and the service as a whole, dual diagnosis practice and a fifth theme of miscellaneous views of the senior staff. The two themes that are the focus of this paper are the effect on service users and effect on staff. These two themes were chosen as this paper is a follow up to [18], which investigated initial impacts of staff using the BDA at a Community Care Unit. The current paper explores the sustained practice change of the implementation of the BDA.

### 3.1. Participant Characteristics

Participants were three senior health professionals in the service. All three participants were female, English-speaking and tertiary-educated: two as nurses and one as an occupational therapist. Two were born in England and one in Australia. The three participants will be referred to as the ‘manager’, the ‘portfolio holder’ and the ‘senior practitioner’.

### 3.2. Effect on Service Users

Many responses were provided within this category with a predominantly positive focus. These responses tended to suggest that the BDA improved engagement between staff and service users, reduced stigma, was more holistic, safe, helped develop treatment goals, promoted service user choice, increased trust between staff and service user, was user friendly and collaborative.

The manager noted that it helped to expand the conversation about particular issues related to drugs. She added that service users felt they had “…a bit more control over what they were doing with their lives” and there was a “… feeling of achievement…” when consumers had engaged in the BDA. It also assisted in changing the way service users perceived the service in that they were “… feeling accepted … that you know they’re not a second-class citizen because they, they use substances…I think just the feeling that they belong somewhere and can talk about it freely without having a negative impact”.

The portfolio holder stated similar positive outcomes from applying the BDA. She stated that: “…implementing the BDA meant that consumers had a more holistic response to all their needs. And that AOD wasn’t excluded from that. That it wasn’t just like we’re just mental health and recognising the link of substance use and mental health and that it’s equally important. So, giving consumers an opportunity to safely explore their substance use and work towards being able to stay in a program around their recovery…”.

She noted that use of the BDA was better than treatment as usual and that it assisted with treatment beyond harm reduction when she stated “… giving consumers a much better opportunity across all their needs”.

Some evidence of the user friendliness of BDA was captured by her comment that “…after they did the first one that they wanted to do a second one”. She also provided evidence of the BDA instrument assisting engagement as “…people felt like … they were taken seriously and that their sense of safety and their sense of wellbeing was respected”. In short, “…they found it helpful”. Her comment that “I think it (doing the BDA) made him (the consumer) sort of feel like ‘oh okay this is about my quality of life, this is about what I want” and “…going through it you know with his preference of which substance he wanted to talk about” demonstrated a level of consumer choice and positive engagement. She further commented on changes to the approach of the service and building trust. She stated “… we can actually stand by the term inclusive (of alcohol and/or other drug use in a mental health setting). Whereas before we couldn’t really. It’s become part of our assessment program…” and “…it sets up from the beginning of their experience in our program that this is how (and) …what we do. This is for everyone …. And I think it does make people feel like they can actually engage or trust the service more”. And “It helps them, you know, yeah, be open…”. Her final comment in relation to effect on consumers was that the BDA instrument makes AOD treatment more mainstream, similar to the way physical health is treated.

As with the other two staff, the senior clinician emphasized the collaborative nature of the BDA instrument. She stated that “…it’s helped more collaboration with clients” because “They do it in partnership (consumers and staff)…”. Similarly, she reported evidence of user friendliness with her comment that “They (consumers) can see the point of it” and that “It’s not like some of the other assessment tools. It’s very practical”. She also stated that “Some people (consumers) have actually taken and kept their copy and have got it with them. I imagine the newest guy we got has actually got that with him. It’s in his handwriting and he’s really taken ownership of it”.

When asked if there were any negative issues associated with using the BDA instrument, the senior clinician replied “No, because it’s such a non-judgemental tool…”. She commented that the BDA “…is something we can use to reduce harm”.

The three participants noted receiving no negative comments from service users in relation to using the BDA, and that they thought service users generally found completing the BDA was a positive experience.

### 3.3. Effect on Staff

This theme explored the senior staff perspectives about the effect that the implementation of the BDA had on staff dual diagnosis practice at the Community Care Unit. Senior staff were asked the question ‘what effect did the implementation of the BDA have on staff’ and their answers were recorded and transcribed. Additional exploratory questions were asked to clarify responses.

#### 3.3.1. The Manager

“I think it gave them (staff) something to structure a conversation which sometimes feels uncomfortable. In relation to using the BDA instrument, staff were saying ‘oh it’s easy to use, you know it’s easy, it’s not difficult’”.

“Also, I think learning about the individual not just about the drug use and its effects. So, learning about that, that individual’s use. The reasons why maybe or what they like about it and it, that exploring … I think really motivated some staff to kind of you know delve a bit further into, into that part of recovery”.

When asked whether the BDA instrument assisted staff engagement with the service user the manager agreed and added “…it’s about the individual, not the use so … it sparked a lot of conversations around interests and different strategies people used”. And that “I think they (Staff) learnt a lot from the individuals (consumers) that they wouldn’t have been aware of”.

Use of the instrument raised conversations about withdrawal more so than treatment as usual. The manager stated “I don’t think that (discussions about withdrawal) was happening as much or as um healthily as it does with the, with the tool”. The use of language that is relevant and appropriate to the consumer was seen as a benefit of the instrument; “Having it (the discussion about harm reduction) in the consumers language so a clinician can understand … the context of it (consumer’s drug use)”.

Use of the instrument promoted knowledge in the generation of harm reduction strategies as “…on both sides there was learning around that (generating harm reduction strategies)” and “(it was a) two way (staff and consumer) learning process”. Interestingly there was mention of how the instrument “…helped that clinician understand more about some of the symptomology that, that drives use”.

The instrument promoted staff thought about other issues also: “It was interesting because people (staff) were sort of talking about, you know, other areas of consumers’ lives where they could maybe think about, you know, certain behaviours that could be minimised through just exploring what was going on before during and after. So yeah it’s interesting to see the lateral thinking that goes on once when someone (staff) is engaged with a tool or with a process”. This promotes more person-centred care [25].

There was some evidence that use of the instrument may have a role in the desire of staff to engage in quality improvement because “… people (staff) really kind of embraced trying some new things”. It also increased confidence as the manager “…could see people feeling more comfortable in approaching the subject (dual diagnosis) with consumers and more comfortable in talking in say clinical review as a forum”. This is evidence of increased staff confidence. Further evidence included a change to the way staff discuss dual diagnosis issues in clinical review: “So it was good to see some really robust conversations happening in clinical review and that definitely came from staff confidence”. Discussion of dual diagnosis issues “… became part of those weekly case management catch up sessions”; this is evidence of sustainability.

Therapeutic alliance and engagement were further enhanced by use of the instrument as the manager stated “…it does strengthen relationships (staff and consumer). Yeah”. She also agreed that the BDA instrument helped increase skills that staff need for their job? “Yeah, yeah definitely”.

She recommended that staff who work in health use the BDA “Absolutely yeah. Now that I’ve moved services I can really notice the difference um in the conversations that happen…”.

In summary she concluded that “I think the more, the more people are aware and if they’re provided with an easy tool and support to use it, it’s so beneficial for staff and the consumer”.

#### 3.3.2. Portfolio Holder

In a manner similar to the manager, the portfolio holder agreed the BDA instrument had “… built their (staff) confidence…” through straightforward training and mentoring. The mentoring was reported to be useful through “…just listening to other people’s experience of using the tool helped build confidence for people (staff) and helped people (staff) learn how to have that conversation”. She also stated that “…staff feel more capable in actually responding to all of you know the consumers’ needs and recognising the importance of actually looking at somebody’s substance use”. She also commented that staff were doing more dual diagnosis training as a result of implementing the BDA.

She also provided evidence of the sustainability of the BDA instrument as “…it’s now part of our (the service’s) assessment program”. And it was written into the assessment template which is a document used with all new referrals to the service.

#### 3.3.3. Senior Clinician

The senior clinician discussed similar themes to both the Manager and Portfolio holders in relation to the role of the instrument in normalising the occurrence of alcohol and/or other drug issues in a mental health service and the need to address it. She said that “Staff have found it difficult to bring up these (dual diagnosis) sorts of conversations with clients so it (the BDA instrument) was a useful way of explaining that even though someone might have a drug or alcohol issue that it wasn’t precluding them to come in to our program”.

She also provided evidence that the BDA instrument assisted in building staff confidence as she stated that “…in terms of confidence about how to do that (dual diagnosis practice) it (the BDA instrument) was a useful thing to focus on. Showing someone that they could disclose that they were using a particular substance. We weren’t going to be judgemental. We were just going to look at how we could reduce you know the harm from that. So, it was a good way to focus really and it gave people a bit more confidence in how to go about that and sort of breaking down goals with clients”.

This prompted a discussion about whether or not the BDA instrument gave staff some form of authority or legitimacy to discuss alcohol and/or other drug use. The Senior Clinician agreed that it did: “Yeah. I think some people just didn’t feel equipped at all. Just another thing they have to (do). I think it did. There was some sort of focus. You’d learnt how to use it and just practicing those skills, so yeah it did I think”.

She stated that the approach taught in the training and mentoring was useful as “It’s the conversational part of it. How do you go about addressing it with clients in a non-judgemental way. That was good. Yeah the approach”. And that “The ones that did the training and the mentoring, it was really useful”. Claiming that “The mentoring part after, the follow up afterwards was really important”.

As per the Manager and the Portfolio Holder, the Senior Clinician didn’t identify any negative impacts of the implementation of the BDA instrument. When this aspect was discussed she replied “Not that I’m aware of”.

The Senior Clinician was asked about whether the BDA instrument contributed to staff thinking about mental health and drug use, dual diagnosis, and she thought it had. She provided the following example: “Yeah. Smoking has come up quite a bit in relation to Clozapine. …And when someone does have a deterioration in mental state we are thinking a bit more around what else is happening in their life. You know like is there an increase in alcohol use?” Further to this she stated “I think it’ (AOD impact on MH) more in focus now” and that “Staff who’ve done the training or have had exposure to that tool (The BDA) are thinking more about that (dual diagnosis)”. This is evidence of a change in work practices to be more dual diagnosis focused.

Overall, she felt that the biggest impact from the implementation of the BDA was an increase in staff confidence to discuss alcohol and/or other drug issues in relation to a person’s mental health issues: “I think the confidence thing is probably the biggest thing and just having some sort of framework. Something you can produce and work with. Staff are interested to learn about the tool”. She further discussed that the BDA instrument “… just fit(ted) so well with … (the service) approach, you know how we work with clients, it is about enabling them to be setting goals around that (alcohol and/or other drug issues) …giving them tools to think about how they can manage because in the end they are only with us for so long”. Given her number of years’ experience it was to some degree surprising to hear her state that “I think as a tool it’s perhaps the one that is the most user-friendly”.

Part of the training includes staff using the BDA instrument on each other. This was seen as a great way for staff to learn to use the BDA. She stated “That’s the best thing you can do when you first use the tool is to try it on yourself”. Unexpectedly, she also discussed that staff had been using the BDA instruments on themselves to gain greater understanding of their own alcohol and/or other drug use issues and that this potentially also “…created more awareness really for us about…and the thing is you know it’s not just about someone with a mental illness you know, it’s everybody isn’t it, we’re all got things we’re grappling with”.

A focus on language that was strengths-based and recovery-focused was a recurring element in conversations with all three participants. All three participants discussed the importance of using strengths-based and recovery-focused language when implementing the BDA instrument. This is important as both mental health and alcohol and/or other drug issues are highly stigmatised conditions. The use of strengths-based and recovery-focused language may serve to reduce stigma [26,27].

## 4. Discussion

The study findings suggest that implementing the BDA harm reduction package has had a positive impact on the service in a number of ways. It is a user-friendly instrument for service users and staff—and therefore more likely to be used by staff. It is useful to use when discussing service users in clinical review. It appears to assist in managing static risk. It has potential for shifting culture and reducing stigma. It assisted with the use of recovery language, and generated positivity amongst staff. It increased staff knowledge and confidence in working with a harm reduction-informed focus. It offered a general health and wellbeing instrument for staff personal use. This change had been sustained for three years at the time of writing.

It supported having a strong therapeutic alliance with service users in mental health settings [28], and keeping service users engaged in treatment is an important part of recovery [29]. Service users with both mental health and illicit drug use, as a dual diagnosis, are at increased risk of disengagement from services, possibly due to a lack of relevant staff training [30]. Service user choice in treatment is important [31] as is staff confidence and the knowledge to apply interventions [32].

### 4.1. Impacts on Ways of Working with Service Users

The perspective of the service may have a large bearing on how dual diagnosis issues are explored. Alcohol and other drug and mental health services may have different perspectives on how best to treat service users with dual diagnosis issues. Mental health services may be more prone to placing more emphasis on the mental health condition and the alcohol and/or other drug service may place more emphasis on the alcohol and/or other drug issue [33].

In addition to this more systemic issue, staff in health and welfare settings are often expected to perform harm reduction without necessarily having a specific instrument with which to do it. They are told what to do but not how or there is an assumed skill that may or may not exist. Without standardised instruments, harm reduction interventions are hard to measure and therefore quality improvement is made all the more complex. Having a specific harm reduction instrument and associated implementation package may be one way to overcome these issues and assist dual diagnosis practice.

The manager reported that “…people (consumers) actually enjoyed the conversation (when using the BDA)” and that “…they (consumers) liked talking about it (harm reduction) rather than just being asked a question and then it not going anywhere you know” [34,35]. We suggest stigma associated with alcohol and/or other drugs contributes to adverse health outcomes, so a tool that can reduce stigma could improve health outcomes or at least not make them worse. Use of the BDA assisted service users in “…a shift in thinking for some service users that maybe, you know, had different experiences in the past”.

### 4.2. The Utility and Benefit of Introducing Clinical Instruments

In health and welfare settings, instruments assist staff to manage complexity by giving staff structure to move forwards with treatment. Tools in general provide a starting point to build new skills that can then be expanded upon. Responses from the three participants reported very similar information in relation to the themes. The clinical significance of these findings is that mental health service users can have their alcohol, caffeine, nicotine and other drug issues explored in a safe supportive, non-judgemental manner. The BDA can assist staff of the mental health service to identify and respond to alcohol and other drug needs, as stipulated in the Victorian Mental health Act 2014.

One of the key issues in dual diagnosis that is often raised is complexity. Having an evidence-based tool that is part of a replicable implementation process may assist in addressing some of this complexity. This opens the door to more sophisticated treatment planning. The BDA has been reported to be user-friendly. Having a clear tool that has been implemented across the service may also assist in staff retention. Use of this tool has further encouraged changes to tenancy agreements, to reflect a more compassionate harm reduction approach, from exclusion to inclusion.

### 4.3. The Manager

The Manager stated “Language is a huge thing. And once people (staff) actually hear themselves and what they’re saying and reframe it, it’s quite powerful”. Similarly, the Portfolio Holder felt that “…role modelling language you know getting rid of some of the stigmatising language that people used about somebody and um you know encouraging people and you know asking questions and framing it in a way that identifies that this is a human being, this is the choice that they’re making and let’s drill down to what actually really matters in this is like what it is for the person”.

When asked if she would recommend implementing the BDA to another service, she replied “Yep. Yeah”. She agreed that implementing the BDA at the service a useful process: “Yes. So I really enjoyed it”. She went on to say, “…having something that was new and fresh in the program um that you can work with long term consumers with and yeah see those outcomes quite clearly within a fairly short amount of time… gave people (staff) a sense of achievement and you know pride in their work that ‘oh what I did actually mattered’”.

### 4.4. Portfolio Holder

The Portfolio Holder stated that she “… kept it on the agenda” and noted the importance of role modelling and having “…these conversations about mental health and alcohol and/or other drug use) and you know we need to overcome our own insecurities around it and just start talking about it”. Part of her role in keeping it on the agenda was about “… reminding people you know like saying like you know ‘oh have they you know been introduced to BDA tool? Is that something they might want to work on?’” and “… challenging the language…” to try and help “… staff feel like they can actually have these conversations in these team meetings and helping the staff know I guess like how to have those conversations that doesn’t end up with ‘you need to just stop’ or you know ‘we need to kick them out, they’re not doing recovery’”. She discussed how there are now other staff that are also taking on this role modelling and encouraging the use of appropriate language. The service is at a point where the Portfolio Holder doesn’t “… need to be the one that is always sort of saying it. Like, you know, I sort of can sit back and just listen to other people do that and feel proud in them but yes, we got there!”.

She also agreed that Implementing the BDA instrument had been a useful process for staff overall? She said “Yep. I do. I think that the BDA you know like yes it’s focused around you know a tool to help you know people with their substance use and kind of plan out what they can do to minimise harm but I feel like it’s actually more for staff”. She went on to say that “…staff having this and it being so simple, like it’s not intimidating…it’s not a document that overwhelms them from the first moment they’re looking at it” so it’s therefore “…it’s more likely to be used…” as “…people (staff) feel comfortable like people (staff) aren’t scared by it…” She stated how “the flow on effect for consumers is that you know they can get so much more out of their stay here. It’s the best outcomes for them rather than ignoring a whole part of this person that influences and impacts every other aspect of who they are”. A further point she made regarding the implementation was that it encouraged staff to be “…interested in, in their professional practice”.

She acknowledged the support from the Manager in “…making such a huge change to the program and that the manager lead by example as she also did the training and mentoring”. In terms of other implementation activities, the Portfolio Holder discussed the creation of a harm reduction information board that staff could interact with and a quiz as being useful.

### 4.5. Implications for Policy and Practice

This study investigated the impact of a harm reduction package on a residential mental health service. The finding was that staff benefit from training in harm reduction, to increase their skills in this area and also to demonstrably offer greater person-centredness and expertise to people using the service, and this should be standard across mental health services.

### 4.6. Future Research

Staff capacity to initiate harm reduction in a consistent manner requires more attention. The Royal Commission into Victoria’s Mental Health System—recommendation 36—notes that dedicated research into mental illness and substance use or addiction is needed, including to ‘support education and training initiatives for a broad range of mental health and alcohol and other drug practitioners and clinicians’ (p. 72) [36]. Further studies could investigate the longitudinal outcomes for service users and staff. A comparative analysis of staff trained in BDA and a control group would be beneficial. Testing the BDA harm reduction package in other settings is also warranted.

### 4.7. Limitations

The harm reduction instrument and package were designed, developed and implemented by one of the study authors. In conducting semi-structured interviews, this could potentially have introduced bias toward the provision of mostly favourable results. However, the advantage of this approach was that the author had intimate knowledge of mental health services and workforce development issues, and was considered a credible interviewer by staff, influencing their willingness to participate and disclose experiences with the instrument and any challenges in implementation.

Findings from a specific, small and purposive sample may not be generalizable to all clinical practice settings aiming to engage in harm reduction treatment planning. There was also a lack of quantitative data, the inclusion of which could have enhanced this type of qualitative study. The impact on service users was reported via relevant clinicians and not through self-report by the service users. The demographics of the three leaders—all were white, females, and tertiary health trained—could be inconsistent with views or experiences in implementation if the cohort were from different culture/language groups. That said, the majority of the mental health clinical workforce in Victoria, and in Australia overall, are indeed white, female and tertiary-educated.

## 5. Conclusions

Workers have found the BDA clinical instrument easy to use with service users, and an effective way to explore alcohol and other drug issues, develop strategies with service users and to set goals. It contributes to overall treatment planning. It serves a number of purposes beyond harm reduction such as risk management, greater staff confidence in talking about alcohol and other drug issues in clinical reviews and building staff confidence to have conversations about other complex issues. It reduces stigma. It may also serve a role as a staff health and wellbeing tool and aid staff retention and job satisfaction. Importantly, these practice changes have been sustained for three years.

## Data Availability

Data are contained within the article.
